# A phase III randomized trial of gantenerumab in prodromal Alzheimer’s disease

**DOI:** 10.1186/s13195-017-0318-y

**Published:** 2017-12-08

**Authors:** Susanne Ostrowitzki, Robert A. Lasser, Ernest Dorflinger, Philip Scheltens, Frederik Barkhof, Tania Nikolcheva, Elizabeth Ashford, Sylvie Retout, Carsten Hofmann, Paul Delmar, Gregory Klein, Mirjana Andjelkovic, Bruno Dubois, Mercè Boada, Kaj Blennow, Luca Santarelli, Paulo Fontoura, for the SCarlet RoAD Investigators

**Affiliations:** 1 Product Development, Neuroscience, Genentech Inc., South San Francisco, CA USA; 2 MedDay Pharmaceuticals, Boston, MA USA; 3 Formerly Roche Translational & Clinical Research Center, New York, NY USA; 4 VU University Medical Center, Amsterdam, The Netherlands; 5 Institute of Neurology, UCL, London, UK; 6 Roche Pharma Research and Early Development, NORD, Basel, Switzerland; 7 Roche Products Limited, Welwyn Garden City, UK; 8 Roche Pharma Research and Early Development, Clinical Pharmacology, Roche Innovation Center, Basel, Switzerland; 9 Clinical Pharmacology and Bioanalytical R&D, Pharmaceutical Sciences, Roche Pharma Research and Early Development, Roche Innovation Center Basel, Basel, Switzerland; 10 Alzheimer Institute and ICM, UMR-S975, Salpêtrière University Hospital, AP-HP, Pierre and Marie Curie University, Paris, France; 11 Research Center and Memory Clinic of Fundació ACE, Institut Català de Neurociències Aplicades, Barcelona, Spain; 12 Research Center and Memory Clinic of Fundació ACE, Institut Català de Neurociències Aplicades, Barcelona, Spain; 13 Formerly Roche Pharma Research and Early Development, NORD, Basel, Switzerland

**Keywords:** Gantenerumab, Alzheimer’s disease, SCarlet RoAD

## Abstract

**Background:**

Gantenerumab is a fully human monoclonal antibody that binds aggregated amyloid-β (Aβ) and removes Aβ plaques by Fc receptor-mediated phagocytosis. In the SCarlet RoAD trial, we assessed the efficacy and safety of gantenerumab in prodromal Alzheimer’s disease (AD).

**Methods:**

In this randomized, double-blind, placebo-controlled phase III study, we investigated gantenerumab over 2 years. Patients were randomized to gantenerumab 105 mg or 225 mg or placebo every 4 weeks by subcutaneous injection. The primary endpoint was the change from baseline to week 104 in Clinical Dementia Rating Sum of Boxes (CDR-SB) score. We evaluated treatment effects on cerebrospinal fluid biomarkers (all patients) and amyloid positron emission tomography (substudy). A futility analysis was performed once 50% of patients completed 2 years of treatment. Safety was assessed in patients who received at least one dose.

**Results:**

Of the 3089 patients screened, 797 were randomized. The study was halted early for futility; dosing was discontinued; and the study was unblinded. No differences between groups in the primary (least squares mean [95% CI] CDR-SB change from baseline 1.60 [1.28, 1.91], 1.69 [1.37, 2.01], and 1.73 [1.42, 2.04] for placebo, gantenerumab 105 mg, and gantenerumab 225 mg, respectively) or secondary clinical endpoints were observed. The incidence of generally asymptomatic amyloid-related imaging abnormalities increased in a dose- and *APOE* ε4 genotype-dependent manner. Exploratory analyses suggested a dose-dependent drug effect on clinical and biomarker endpoints.

**Conclusions:**

The study was stopped early for futility, but dose-dependent effects observed in exploratory analyses on select clinical and biomarker endpoints suggest that higher dosing with gantenerumab may be necessary to achieve clinical efficacy.

**Trial registration:**

ClinicalTrials.gov, NCT01224106. Registered on October 14, 2010.

**Electronic supplementary material:**

The online version of this article (doi:10.1186/s13195-017-0318-y) contains supplementary material, which is available to authorized users.

## Background

Alzheimer’s disease (AD) is characterized by the presence of amyloid plaques and neurofibrillary tangles in the brain. Amyloid plaques are composed primarily of aggregated amyloid-beta (Aβ) peptide, which is deposited in the brain parenchyma, likely decades before clinical symptoms manifest [1]. Although the accumulation of presumably neurotoxic, aggregated Aβ is at the core of the amyloid hypothesis of AD [2], the specific process by which Aβ may lead to neuronal death remains unclear. Clinical progression has been reported to follow a predictable pattern, with patients transitioning through a preclinical stage, followed by a prodromal stage characterized by biomarker findings indicative of accumulating disease burden and cognitive symptoms not of sufficient severity to impact patient functioning, and eventually reaching the dementia stage.

Prior to the initiation of the SCarlet RoAD study (NCT01224106; WN25203), researchers in most clinical trials identified patients with mild cognitive impairment (MCI) using clinical criteria, such as an episodic memory deficit or Clinical Dementia Rating (CDR) global score. Heterogeneity of patients with MCI has consistently been identified as a factor hampering the possibility of identifying a clinical benefit [3]. Subsequently, research criteria that included biomarkers of AD pathology were developed in an attempt to more specifically identify patients in the prodromal phase of AD [4, 5]. Because cerebrospinal fluid (CSF) levels of Aβ_42_ have been found to be low in prodromal AD [6], CSF testing for low Aβ_42_ levels was required for inclusion in the SCarlet RoAD study.

Gantenerumab (RO4909832, RG1450) is a human anti-Aβ monoclonal antibody that binds with high affinity to aggregated Aβ and promotes its removal by Fc receptor-mediated phagocytosis [7]. In a phase I study of 16 patients with mild to moderate AD, gantenerumab treatment resulted in a rapid reduction in brain amyloid load over the course of 6 months [8]. Gantenerumab intravenous (IV) doses of 60 mg (equivalent to ~ 100 mg subcutaneous [SC]) and 200 mg (equivalent to ~ 330 mg SC) given every 4 weeks were associated with dose-dependent cortical amyloid standardized uptake value ratio (SUVr) reduction [8] compared with placebo. At 200 mg IV, *apolipoprotein E (APOE*) ε4 genotype-dependent vasogenic edema (amyloid-related imaging abnormalities [ARIA]-E) and amyloid-related imaging abnormality microbleeds (ARIA-H) were detected, suggesting an optimal target dose for the investigation of efficacy above 100 mg but below 330 mg SC. Accordingly, gantenerumab SC doses of 105 mg and 225 mg given every 4 weeks were selected for the SCarlet RoAD study, with *APOE* ε4 homozygous patients randomized to the lower dose or placebo only. SCarlet RoAD was the first phase III study in prodromal AD confirmed by CSF amyloid analysis with a single primary endpoint, the CDR Sum of Boxes (CDR-SB). The primary objective was to evaluate the effect of gantenerumab (105 and 225 mg) compared with placebo on CDR-SB in prodromal AD over the course of 2 years of treatment. In this paper, we report the efficacy, biomarker, and safety data available at the time of SCarlet RoAD futility analysis, including results of preplanned and exploratory analyses.

## Methods

SCarlet RoAD was a phase III, multicenter, randomized, double-blind, placebo-controlled, parallel-group, 2-year study of gantenerumab in prodromal AD. The study was conducted globally across 128 sites. SCarlet RoAD was approved by individual institutional ethics committees or institutional review boards and was conducted in accordance with the principles of the Declaration of Helsinki and good clinical practice (GCP). Written informed consent was obtained from each patient.

### Patients

A total of 3089 patients were screened for eligibility for this study. The screening period lasted up to 8 weeks, with rescreening permitted after ≥ 3 months for patients who did not meet select eligibility criteria. It was recommended that the first screening tests be the CDR, Free and Cued Selective Reminding Test (FCSRT), and Mini Mental State Examination (MMSE), in any order. CSF collection and magnetic resonance imaging (MRI) were conducted only after neuropsychological, electrocardiographic, and laboratory tests confirmed eligibility. Participation in an exploratory amyloid positron emission tomography (PET) substudy was optional and was not available at all sites. For patients in this substudy, a positive screening scan for amyloid PET was required and was performed once eligibility for the main study had been confirmed.

Patients in SCarlet RoAD were 50–85 years of age and met International Working Group criteria for prodromal AD [4], with biomarker evidence of amyloid pathology and largely preserved functional abilities such that a diagnosis of dementia could not be made. Clinical status was documented by an MMSE score ≥ 24, a CDR global score of 0.5 with an accompanying memory box score of 0.5 or 1.0, abnormal memory function based on an FCSRT score of either < 17 free recall, < 40 total recall, or < 20 free recall, and < 42 total recall; a score ≤ 4 on the modified Hachinski Ischemic Scale [9]; and absence of depression documented by a score ≤ 6 on the Geriatric Depression Scale (GDS). Evidence of amyloid pathology was required as determined by a CSF Aβ_1–42_ level ≤ 600 ng/L (INNOTEST^®^ Aβ_1–42_; Fujirebio, Ghent, Belgium).

Exclusion criteria were neurological disease other than AD, abnormal brain MRI at screening (including three or more microhemorrhages [1.5 T], two or more lacunar infarcts, extensive/confluent deep white matter lesions, or any space occupying lesions), a major psychiatric disorder, and a history of stroke or any clinically unstable medical illness. Symptomatic treatment with memantine or acetylcholinesterase inhibitors was not permitted at any time during the study; patients requiring such antidementia therapy were to be discontinued from the study.

### Randomization and masking

The planned sample size was 770, with patients meeting eligibility criteria randomized to treatment with placebo, gantenerumab 105 mg SC, or gantenerumab 225 mg SC every 4 weeks (ratio 2:1 for active placebo). Patients with zero or one *APOE* ε4 allele were randomized to any treatment group, whereas *APOE* ε4 homozygotes could be randomized only to placebo or gantenerumab 105 mg SC. *APOE* ε4 status was blinded to sponsor, patient, and investigator. The algorithm for dynamic patient allocation to treatment was based on minimization with biased coin assignment. The randomization was stratified by PET substudy participation (participation vs nonparticipation), *APOE* ε4 allele status (0 ε4 vs 1 ε4 vs 2 ε4), and region (Europe vs rest of world). The study was conducted in a double-blind manner. A potentially unblinded person was involved in the preparation of study medication but was not involved with patient care.

### Procedures

Treatment with placebo, gantenerumab 105 mg, or gantenerumab 225 mg was administered SC every 4 weeks in the abdomen. The gantenerumab drug product was manufactured by Roche Pharma AG (Grenzach-Wyhlen, Germany) in accordance with Roche standards and local regulations.

### Clinical outcomes

The primary endpoint was the change from baseline in CDR-SB at week 104 [10]. Secondary endpoints included changes in cognition, behavior, and daily function over 104 weeks. Cognition was assessed using the 13-item Alzheimer’s Disease Assessment Scale–Cognitive subscale (ADAS-Cog 13), the MMSE, a computerized cognitive battery (Cambridge Neuropsychological Test Automated Battery [CANTAB]), and the FCSRT. Behavior and daily functioning were assessed using the Neuropsychiatric Inventory Questionnaire (NPI-Q) and the Functional Activities Questionnaire (FAQ), respectively. Clinical assessments were carried out before the first dose and every 12 weeks thereafter.

### Biomarker outcomes

Biomarker assessments included amyloid PET, brain volumes as measured by MRI, CSF concentrations of Aβ_1–42_, total tau (t-tau), phosphorylated tau 181 (p-tau), and neurogranin.

#### Amyloid PET

Participants in the [^18^F]florbetapir substudy underwent PET scanning at baseline and at weeks 20, 60, and 100. PET acquisition started 50 minutes postinjection, and three 5-minute frames were acquired. All PET data were corrected for radioactive decay, scatter, and attenuation. The data were also assessed for artefacts and patient motion. All further PET data processing was performed in PMOD (PMOD Technologies, Zürich, Switzerland). After motion correction and averaging of the three frames, the PET scan was coregistered to the cropped, skull-stripped screening MRI and normalized to the Montreal Neurological Institute space using the normalization parameters obtained from the MRI scan. The [^18^F]florbetapir global cortical signal was calculated as the volume-weighted, gray matter-masked average SUVr [11] of five bilateral cortical regions defined on the basis of Automated Anatomical Labeling template: anterior and posterior cingulate cortex, parietal, lateral temporal, and frontal cortex [12], using cerebellar cortex as the reference region for intensity normalization. Other reference regions investigated included whole cerebellum and pons.

#### MRI

MRI was performed using 1.5-T magnets. In exceptional circumstances, a 3-T magnet was accepted. MRI scans were obtained for subject screening and safety monitoring and to determine potential treatment effects on brain volumes. Each time an MRI scan was scheduled, we acquired near-isotropic 3D T1-weighted gradient echo, axial 5-mm T2*-weighted gradient echo with a minimum echo time of 20 milliseconds; T2-weighted spin-echo fluid-attenuated inversion recovery; and, if available, diffusion-weighted scans. Images were centrally quality-controlled and read by a neuroradiologist. Screening, week 48, and week 104 MRI scans were used for volumetric analysis of hippocampi, whole brain, and ventricles [13, 14]. Hippocampal volume was assessed by trained technicians using manual tracing. Whole-brain volume change was measured using the *k*-means brain boundary shift integral [14]. Ventricular volume change was measured using the ventricular boundary shift integral [13].

#### CSF

CSF was sampled at screening and at weeks 52 (optional) and 104. Screening samples were analyzed using INNOTEST® Aβ_1–42_ for eligibility assessment. For longitudinal analyses, CSF biomarkers from all time points were measured using the Elecsys® β-Amyloid_1–42_, t-tau, and p-tau_181P_ immunoassays (Roche Diagnostics GmbH, Rotkreuz, Switzerland) [15]. Neurogranin, a biomarker associated with synaptic dysfunction and degeneration in AD [16, 17], was evaluated at the clinical neurochemistry laboratory of Dr. Kaj Blennow, Gothenburg, Sweden, using an in-house enzyme-linked immunosorbent assay [18].

### Safety monitoring

Safety was evaluated by reports of adverse events, clinical laboratory testing (hematologic and serum chemistry, and urinalysis), vital sign assessments, physical and neurological examinations, electrocardiography, antidrug antibody titers, and brain MRI. MRI reads documented all ARIA-E and ARIA-H, as well as scoring of ARIA-E using the ARIA-E rating scale (scale range 0–60, with higher scores indicating greater severity) [19]. All ARIA were recorded as adverse events and additionally reviewed by an independent MRI committee, whose reports were shared regularly with the independent data monitoring committee (IDMC). Patients were queried about new central nervous system symptoms following the identification of an ARIA. Upon new ARIA-E findings, treatment was withheld. MRI was performed approximately every 4–8 weeks until the ARIA-E had resolved or clearly decreased and stabilized, upon which, treatment ensued at half the original dose. If a second ARIA-E occurred, treatment was permanently discontinued.

Treatment modification and discontinuation due to ARIA-H was based on cumulative numbers of new events over a 12-month period: if more than four events, treatment was discontinued; if more than two (but less than or equal to four), the dose was halved. For patients on half-dose, treatment was to be discontinued if new ARIA-H events were greater than two since the dose was halved or within the past 12 months, whichever was shorter. Depressive and suicidal symptoms were assessed using the GDS and the Columbia Suicide Severity Rating Scale.

### Statistical analysis

The sample size was calculated to allow ≥ 80% power to demonstrate an effect size of 0.35 on the primary endpoint (gantenerumab 225 mg vs placebo) at the type I error level of *p* ≤ 0.05 (two-sided). Power calculations were based on simulations of the mixed-effects model repeated measurement (MMRM) analysis planned for statistical assessment of the primary efficacy variable.

The primary efficacy outcome—mean change from baseline on CDR-SB at week 104—was assessed using MMRM analysis incorporating data up to 104 weeks of treatment. The model included the change from baseline in the CDR-SB score as the dependent variable. The effects in the model included independent variables of the fixed categorical effects of treatment, assessment weeks relative to the first dose of study medication (i.e., time), treatment-by-time interaction, and *APOE* ε4 status (carrier vs noncarrier), along with continuous covariates of the baseline CDR-SB value and hippocampal volume at baseline. Time was treated as the repeated variable within a subject. Subject, treatment, and time were treated as class variables. An unstructured variance-covariance structure was applied to model the within-subject errors. The primary MMRM was applied to the CDR-SB, ADAS-Cog 13, MMSE, and FAQ endpoints, as well as other endpoints, including CANTAB, FCSRT, and NPI-Q. Change from baseline in PET SUVr was evaluated using an analysis of covariance model with baseline value and treatment as independent variables. A nonparametric Wilcoxon test was used for percentage change from baseline in CSF biomarkers to minimize the influence of potential outliers. All reported exploratory *p* values refer to comparisons between placebo and each gantenerumab arm. Comparisons of the gantenerumab 225 mg and placebo arms did not include *APOE* ε4 homozygote patients. The analysis population included all study participants who received at least one injection of study medication.

An IDMC served as an independent group to examine all safety and efficacy data on a roughly quarterly basis. Rules for the preplanned futility analysis (50% patients reaching 2-year endpoint) were such that the IDMC would recommend study termination if the likelihood of achieving an effect size of 0.2 at the end of the study was < 15% based on estimates from the prespecified MMRM analysis model for the primary endpoint.

### Role of the funding source

The sponsor designed the study in consultation with the academic authors. Data were gathered by the study investigators, analyzed by the sponsor, and interpreted in collaboration with the academic authors. All authors were involved in the development of the manuscript. The academic authors had full access to the study data and vouch for the accuracy and integrity of the data and the fidelity of this report to the study protocols.

## Results

### Study population

**Fig. 1:**
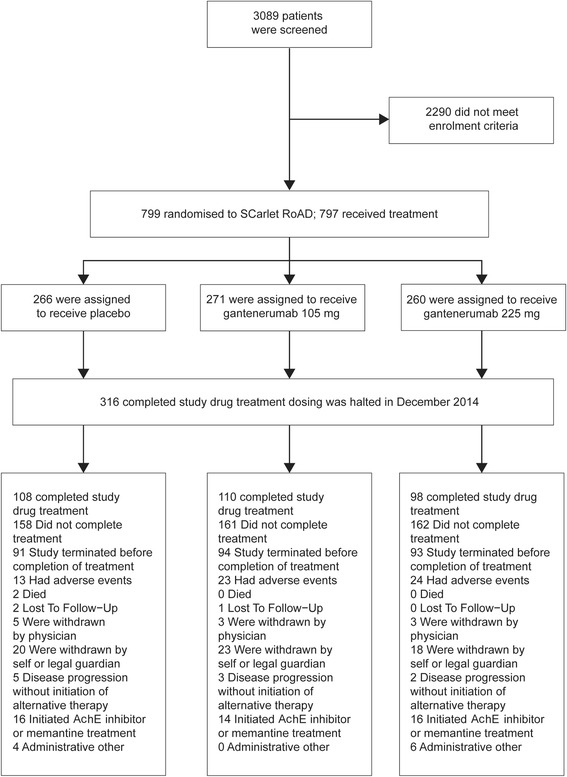
Enrollment, randomization, and 2-year completion in the SCarlet RoAD study. *AChE* Acetylcholinesterase

Of 3089 screened patients, 797 (25.9%) were randomized and received at least one injection of study medication. At the time of the interim analysis, 316 had completed 2 years of treatment. An additional 278 were enrolled but had not completed 2 years of treatment, and 203 patients had discontinued treatment, with the most common reasons being adverse events, self- or legal guardian withdrawal of consent, or the initiation of symptomatic therapies (Fig. 1).

### Baseline characteristics

There were no differences between the treatment arms with respect to age, sex, race, education, or weight (Table 1). By design, there were no patients homozygous for *APOE* ε4 in the gantenerumab 225 mg arm. There were no differences between treatment groups with respect to baseline clinical scores or CSF biomarkers (Table 1).

**Table 1:** Baseline characteristics of patients in the SCarlet RoAD study

Variable	Intention-to-treat population (*n* = 797)		
	Placebo (*n* = 266)	Gantenerumab 105 mg (*n* = 271)	Gantenerumab 225 mg (*n* = 260)
Age, years, mean (SD)	69.5 (7.5)	70.3 (7.0)	71.3 (7.1)
Education, years, mean (SD)	89.8%	93.0%	91.9%
Weight, kg, mean (SD)	12.6 (4.3)	12.9 (4.8)	12.1 (4.5)
*APOE* ε4 genotype, %^a^	69.8 (12.9)	70.5 (13.6)	70.1 (12.5)
0ε4	29.7%	21.0%	38.5%
1ε4	50.4%	41.0%	61.5%
2ε4	19.9%	38.0%	–
Clinical scores			
CDR-SB, mean score (SD)	2.1 (1.0)	2.2 (1.0)	2.0 (0.9)
ADAS-Cog 13, mean score (SD)	23.5 (7.2)	23.1 (6.9)	23.0 (6.2)
FAQ, mean score (SD)	4.9 (4.3)	4.6 (3.9)	4.8 (4.3)
FCSRT-Total Recall, mean score (SD)	29.3 (10.8)	28.3 (10.8)	30.5 (10.4)
MMSE, mean score (SD)	25.7 (2.1)	25.7 (2.3)	25.7 (2.2)
CSF biomarkers			
Aβ_42_, pg/ml, mean (SD)	487.8 (170.4)	475.3 (142.2)	511.8 (172.0)
t-tau, pg/ml, mean (SD)	556.3 (203.8)	563.2 (239.1)	544.5 (220.5)
p-tau, pg/ml, mean (SD)	84.0 (31.4)	86.3 (39.5)	82.5 (34.2)
Neurogranin, pg/ml, mean (SD)	474.8 (260.7)	500.5 (270.0)	484.9 (293.9)

*Abbreviations: ADAS-Cog* Alzheimer’s Disease Assessment Scale–Cognitive subscale, *APOE* Apolipoprotein E, *CDR-SB* Clinical Dementia Rating Sum of Boxes, *CSF* Cerebrospinal fluid, *FAQ* Functional Activities Questionnaire, *FCSRT* Free and Cued Selective Reminding Test, *MMSE* Mini Mental State Examination

^a^By design, there were no *APOE* 2ε4 patients in the gantenerumab 225 mg arm

The baseline demographics and disease characteristics of patients in the PET substudy and those of patients with CSF biomarker data at week 104 did not substantially differ from the overall study population and were well balanced across treatment arms (data not shown).

### Interim analysis

At the preplanned interim analysis, the prespecified stopping criterion for futility was met. With an estimated value of 6%, the predictive probability of success was below the prespecified cutoff of 15%. There was no difference between the placebo arm and either of the gantenerumab treatment arms in the primary endpoint (change from baseline in CDR-SB score at 2 years). The IDMC recommended terminating the trial for futility based on a lack of efficacy and unrelated to safety findings or concerns. This recommendation was endorsed by the sponsor’s review board. Following this interim analysis, dosing of study drug was halted, and the study was unblinded. Data collected up to the time of unblinding are presented here.

Therefore, all analyses presented below, including *p* values, should be considered as strictly descriptive and exploratory. In this context, no formal *p* value correction for multiple testing was applied, but multiplicity was considered in the interpretation of results.

### Exploratory efficacy analysis

**Fig. 2:**
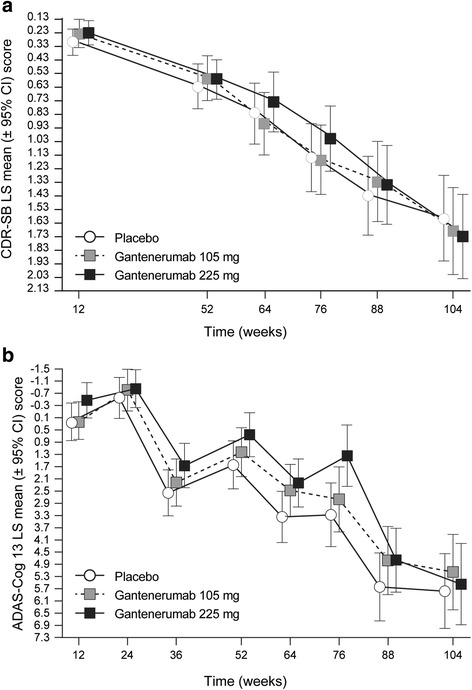
Least squares mean (±95% CI) change from baseline in CDR-SB (**a**) and ADAS-Cog 13 score (**b**). *CDR-SB* Clinical Dementia Rating Sum of Boxes, *ADAS-Cog 13* Alzheimer’s Disease Assessment Scale–Cognitive subscale, *LS* Least squares

In addition to the primary endpoint (CDR-SB), the analysis of secondary cognitive, functional, and behavioral endpoints also showed no treatment effect at 2 years (Table 2, Fig. 2a and b), which is further illustrated by all exploratory *p* values from the MMRM applied to CDR-SB, ADAS-Cog 13, MMSE, and FAQ being > 0.05 (Table 2). FCSRT with Immediate Recall total recall, CANTAB, and NPI-Q data showed no treatment effect (data not shown).

**Table 2:** Least squares mean change in primary and secondary clinical outcomes in mixed-effects model repeated measurement statistical analysis

At week 104	Placebo	Gantenerumab 105 mg		Gantenerumab 225 mg	
	LS mean (95% CI)	LS mean (95% CI)	*p* Value vs placebo	LS mean (95% CI)	*p* Value vs placebo
Primary endpoint					
CDR-SB					
Change from baseline	1.60 (1.28, 1.91)	1.69 (1.37, 2.01)	–	1.73 (1.42, 2.04)	–
Difference from placebo	–	0.10 (−0.35, 0.54)	0.67	0.18 (−0.28, 0.63)	0.45
Secondary endpoint					
ADAS-Cog 13					
Change from baseline	5.77 (4.54, 6.99)	5.14 (3.91, 6.38)	–	5.54 (4.21, 6.87)	–
Difference from placebo	–	−0.62 (−2.34, 1.09)	0.48	−0.27 (−2.23, 1.70)	0.79
FAQ					
Change from baseline	4.70 (3.71, 5.68)	5.93 (4.93, 6.93)	–	4.57 (3.58, 5.55)	–
Difference from placebo	–	1.23 (−0.16, 2.62)	0.08	−0.27 (−1.72, 1.18)	0.72
MMSE					
Change from baseline	−2.93 (−3.50, −2.35)	−3.02 (−3.60, −2.44)	–	−2.73 (−3.33, −2.14)	–
Difference from placebo	–	−0.10 (−0.90, 0.71)	0.81	0.34 (−0.54, 1.22)	0.45

*Abbreviations: ADAS-Cog 13* Alzheimer’s Disease Assessment Scale–Cognitive subscale, *CDR-SB* Clinical Dementia Rating Sum of Boxes, *FAQ* Functional Activities Questionnaire, *LS* Least squares, *MMSE* Mini Mental State Examination

### Exploratory biomarker analysis

Exploratory biomarker analyses suggested a dose-dependent effect of gantenerumab on brain amyloid load as measured by PET, as well as on downstream biomarkers of neural and synaptic degeneration (CSF tau species and neurogranin).

#### Amyloid PET

**Fig. 3:**
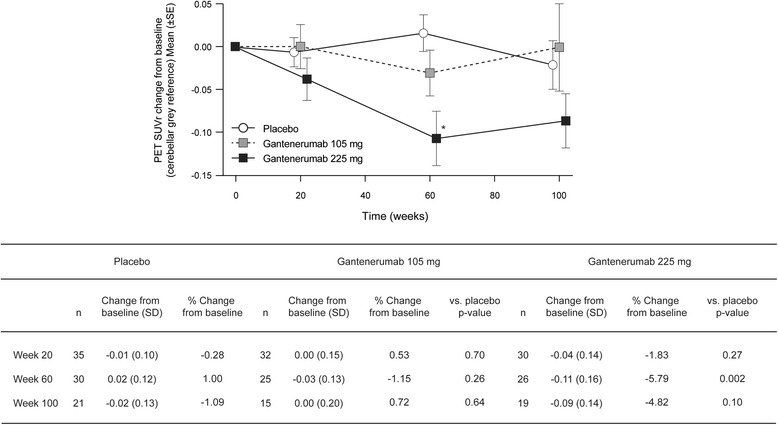
Mean (±SE) change from baseline in PET SUVr (cerebellar *gray* reference). *p < 0.01 vs placebo. *PET SUVr* Positron emission tomography standardized uptake value ratio

There were 115 subjects with PET data at baseline, of whom 55 had PET data at the end of 2 years of study treatment. The mean cortical composite PET SUVr at baseline using the prespecified mean cerebellar gray reference region was 1.68, 1.65, and 1.62 for the placebo, 105-mg, and 225-mg dose groups, respectively. The effect of gantenerumab on PET SUVr was dose- and time-dependent (Fig. 3). PET SUVr was reduced from baseline by an average 4.8% (absolute mean difference −0.09, *p* = 0.1 vs placebo) at week 100 in the 225-mg dose group. There was no effect on brain amyloid load in either the gantenerumab 105-mg dose group (0.72% change from baseline, absolute mean difference 0.00) or the placebo group (1.09% change from baseline, absolute mean difference −0.02) (Fig. 3). Exploratory analyses using alternative reference regions support that the drug-placebo difference at the 225-mg dose level was robust (data not shown).

#### MRI volumetry

No difference between treatment arms at either of the doses tested was seen for any volumetric MRI measure investigated: whole brain, left/right hippocampal, and ventricular volume (data not shown).

#### CSF

**Fig. 4:**
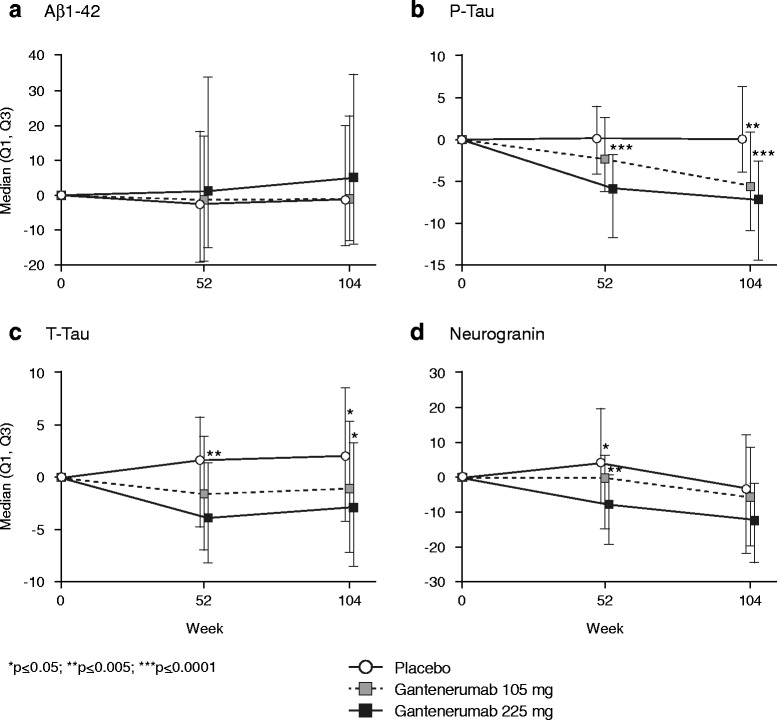
Percentage changes in median cerebrospinal fluid biomarker levels for Aβ_1–42_, p-tau, t-tau, and neurogranin. *p ≤ 0.05; **p ≤ 0.005; ***p ≤ 0.0001. *Aβ* Amyloid-beta, *p-tau* Phosphorylated tau 181

CSF samples were not collected from all individuals postbaseline. Statistical analysis was performed on samples from patients who had at least one postbaseline CSF sample (*n* = 209). For neurogranin, the numbers were lower because of assay performance; that is, data from patients with neurogranin levels below the limit of quantification were excluded. Results are summarized in Table 3. Results suggested dose- and time-dependent reductions in t-tau and p-tau (Fig. 4). At week 104, significantly greater reductions from baseline in the gantenerumab 105 mg and 225 mg treatment arms compared with the placebo arm were observed for CSF p-tau (*p* ≤ 0.001 and *p* ≤ 0.001, respectively) and t-tau (*p* = 0.05 and *p* = 0.02, respectively). The results also suggested a reduction of CSF neurogranin at the higher dose (Table 3, Fig. 4). No significant change in the levels of CSF Aβ_42_ over 2 years of gantenerumab treatment compared with placebo were observed, even though a numerical increase was seen with gantenerumab 225 mg at week 104 only.

**Table 3:** Cerebrospinal fluid biomarker findings

Change from baseline at week 104	Placebo	Gantenerumab 105 mg		Gantenerumab 225 mg	
	Change (%), median (Q1, Q3)	Change (%), median (Q1, Q3)	*p* Value vs placebo	Change (%), median (Q1, Q3)	*p* Value vs placebo
Aβ_42_, pg/ml	*n* = 72 −0.85% (−15.31, 21.03)	*n* = 71 −1.06% (−19.33, 25.88)	0.98	*n* = 66 7.55% (−13.96, 35.09)	0.09
t-tau, pg/ml	*n* = 72 2.04% (−4.56, 8.72)	*n* = 71 −1.08% (−7.65, 5.86)	0.05	*n* = 66 −2.91% (−8.54, 3.40)	0.02
p-tau, pg/ml	*n* = 72 0.08% (−3.91, 6.49)	*n* = 71 −5.61% (−11.07, 0.97)	≤ 0.001	*n* = 66 −7.15% (−14.48, −2.41)	≤ 0.001
Neurogranin, pg/ml	*n* = 65 −3.24% (−20.64, 12.27)	*n* = 66 −4.58% (−19.89, 10.13)	0.79	*n* = 63 −11.76% (−23.69, 6.48)	0.18

*Abbreviations: Aβ* Amyloid-beta, *p-tau* Phosphorylated tau, *t-tau* Total tau

### Further exploratory analyses

Despite the results of the futility analysis, the presence of a drug effect on biomarkers of target engagement and neurodegeneration, as described above, triggered further exploratory analyses to gain understanding of the results of the study and to potentially guide future development of gantenerumab. An aspect of this analysis was to explore the impact of the observed rate of disease progression in the prodromal AD population. These analyses were limited to 2-year completers.

Before the SCarlet RoAD study was unblinded, an AD progression model was built for CDR-SB score using the Alzheimer’s Disease Neuroimaging Initiative (ADNI) database (http://adni.loni.usc.edu/) [20]. The model distinguishes two population types: “slow” progressing and “fast” progressing. Further statistical investigations over a large series of potential covariates at baseline determined CDR-SB, FAQ, and hippocampal volume to be the three main factors in predicting progression type and a prognostic “signature” was therefore proposed as a method to predict “fast” or “slow” progression of disease for patients with prodromal AD.

Applying this algorithm to the SCarlet RoAD 2-year completer population (baseline data), almost two-thirds of the trial population were predicted to be slow progressors (*n* = 202), and one-third could be designated as fast progressors (*n* = 108) (Table 4). By design, fast and slow progressors have slightly different mean baseline characteristics, with fast progressors having higher baseline FAQ and CDR-SB and smaller hippocampal volume. Fast progressors also included fewer *APOE* ε4 noncarriers. However, within the fast and slow subgroups, baseline characteristics appeared well balanced between the treatment arms (data not shown). Given the small sample size in the subgroups of this exploratory analysis, it was deemed that median and IQR data would be adequate summary statistics to report results.

**Table 4:** Fast and slow progressors in the SCarlet RoAD population

Variable, median score (Q1; Q3)	Change from baseline at week 104 (*n* = 310^a^)					
	Slow progressors (*n* = 202)			Fast progressors (*n* = 108)		
	Placebo (*n* = 70)	Gantenerumab 105 mg (*n* = 57)	Gantenerumab 225 mg (*n* = 75)	Placebo (*n* = 35)	Gantenerumab 105 mg (*n* = 47)	Gantenerumab 225 mg (*n* = 26)
Primary endpoint						
CDR-SB	0.5 (0, 1.5)	0.5 (0, 2)	1.0 (0, 1.5)	1.5 (0.5, 3)	1.0 (0, 2.75)	2.0 (1, 2.88)
Secondary endpoints						
ADAS-Cog 13	3.34 (−1.41, 8.41)	3.5 (−2.5, 6.25)	3.33 (−0.34, 8.67)	6.0 (2.34, 12.17)	4.84 (1.5, 7.92)	2.66 (0.67, 7.5)
CANTAB	−1.43 (−2.52, −0.31)	−1.14 (−3, 0.97)	−0.99 (−3.22, 0.56)	−2.42 (−4.09, 0.07)	−1.31 (−3.27, 0.25)	−0.81 (−1.98, 0.69)
FAQ	1 (0, 5)	1 (0, 7)	2 (0, 6)	5 (2.5, 8)	6 (2, 8.5)	4 (1, 9)
MMSE	−1 (−4, 0)	−1 (−4, 0.25)	−2 (−3, 0)	−3.5 (−4.75, −2)	−3 (−4.5, 0)	−2 (−4, 0)

*Abbreviations: ADAS-Cog 13* Alzheimer’s Disease Assessment Scale–Cognitive subscale, *CANTAB* Cambridge Neuropsychological Test Automated Battery, *CDR-SB* Clinical Dementia Rating Sum of Boxes, *FAQ* Functional Activities Questionnaire, *MMSE* Mini Mental State Examination

^a^Six patients completing study drug treatment had missing efficacy assessment at the week 104 visit time window

In the fast progressor subgroup, results suggested a dose-dependent slowing of decline in ADAS-Cog 13, CANTAB, and MMSE, but not in CDR-SB score change, from baseline at 2 years (Table 4). The slow progressor subgroup did not show any difference in decline across treatment groups.

### Adverse events

No notable differences were seen in the rate of serious adverse events and death across groups (Table 5). Two adverse event terms were reported in gantenerumab-treated patients at a rate of > 5% and twofold greater than in the placebo arm: injection site erythema and ARIA (Table 6).

**Table 5:** Summary of adverse events

Event	Safety evaluable population (*n* = 797)		
	Placebo (*n* = 266)	Gantenerumab 105 mg (*n* = 271)	Gantenerumab 225 mg (*n* = 260)
Any adverse event	250 (94.0%)	241 (88.9%)	240 (92.3%)
Any serious adverse event	55 (20.7%)	48 (17.7%)	46 (17.7%)
Any death	6 (2.3%)	0	2 (0.8%)
Cardiac disorders	24 (9.0%)	26 (9.6%)	22 (8.5%)
Ear and labyrinth disorders	11 (4.1%)	15 (5.5%)	13 (5.0%)
Eye disorders	20 (7.5%)	16 (5.9%)	23 (8.8%)
Gastrointestinal disorders	65 (24.4%)	64 (23.6%)	63 (24.2%)
Diarrhea	14 (5.3%)	15 (5.5%)	15 (5.8%)
General disorders and administration site conditions	44 (16.5%)	78 (28.8%)	90 (34.6%)
Injection site erythema	3 (1.1%)	29 (10.7%)	35 (13.5%)
Fatigue	8 (3.0%)	7 (2.6%)	15 (5.8%)
Infections and infestations	110 (41.4%)	110 (40.6%)	119 (45.8%)
Nasopharyngitis	17 (6.4%)	30 (11.1%)	20 (7.7%)
Urinary tract infection	26 (9.8%)	16 (5.9%)	22 (8.5%)
Upper respiratory tract infection	11 (4.1%)	13 (4.8%)	18 (6.9%)
Influenza	13 (4.9%)	13 (4.8%)	15 (5.8%)
Bronchitis	10 (3.8%)	10 (3.7%)	14 (5.4%)
Injury, poisoning, and procedural complications	73 (27.4%)	65 (24.0%)	59 (22.7%)
Fall	28 (10.5%)	23 (8.5%)	28 (10.8%)
Investigations	40 (15.0%)	35 (12.9%)	48 (18.5%)
Metabolism and nutrition disorders	23 (8.6%)	21 (7.7%)	24 (9.2%)
Musculoskeletal and connective tissue disorders	82 (30.8%)	72 (26.6%)	72 (27.7%)
Back pain	26 (9.8%)	16 (5.9%)	25 (9.6%)
Arthralgia	20 (7.5%)	12 (4.4%)	16 (6.2%)
Musculoskeletal pain	15 (5.6%)	6 (2.2%)	5 (1.9%)
Neoplasms benign, malignant, and unspecified (including cysts and polyps)	20 (7.5%)	16 (5.9%)	21 (8.1%)
Nervous system disorders	123 (46.2%)	126 (46.5%)	127 (48.8%)
Headache	36 (13.5%)	34 (12.5%)	25 (9.6%)
Dizziness	21 (7.9%)	21 (7.7%)	27 (10.4%)
Psychiatric disorders	76 (28.6%)	65 (24.0)	73 (28.1%)
Depression	14 (5.3%)	23 (8.5%)	25 (9.6%)
Anxiety	19 (7.1%)	20 (7.4%)	16 (6.2%)
Renal and urinary disorders	19 (7.1%)	21 (7.7%)	22 (8.5%)
Reproductive system and breast disorders	12 (4.5%)	14 (5.2%)	15 (5.8%)
Respiratory, thoracic, and mediastinal disorders	32 (12.0%)	29 (10.7%)	33 (12.7%)
Skin and subcutaneous tissue disorders	31 (11.7%)	39 (14.4%)	39 (15.0%)
Surgical and medical procedures	21 (7.9%)	18 (6.6%)	18 (6.9%)
Vascular disorders	33 (12.4%)	19 (7.0%)	30 (11.5%)
Hypertension	18 (6.8%)	11 (4.1%)	19 (7.3%)

Events with an incidence of at least 5% in any treatment group are shown

**Table 6:** Amyloid-related imaging abnormality findings by *APOE* ε4 genotype and severity of amyloid-related imaging abnormalities-vasogenic edema events

	Placebo (*n* = 266)	Gantenerumab 105 mg (*n* = 271)	Gantenerumab 225 mg (*n* = 260)
ARIA-E	2 (0.8%)	18 (6.6%)	35 (13.5%)^a^
0ε4 patients	2 (2.5%)	1 (1.8%)	11 (11.0%)
1ε4 patients	0	6 (5.4%)	24 (15.0%)
2ε4 patients	0	11 (10.7%)	–
Maximum severity of ARIA–E, mean (±SD)^b^	4.0 (4.2)	4.0 (2.1)	5.7 (6.9)
ARIA-H	35 (13.2%)	62 (22.9%)	42 (16.2%)^a^
0ε4 patients	4 (5.1%)	7 (12.3%)	11 (11.0%)
1ε4 patients	19 (14.2%)	22 (19.8%)	31 (19.4%)
2ε4 patients	12 (22.6%)	33 (32.0%)	–

*ARIA-E* Amyloid-related imaging abnormalities-vasogenic edema, *ARIA-H* Amyloid-related imaging abnormalities-hemosiderin

^a^By design, there were no *APOE* 2ε4 patients in the gantenerumab 225 mg arm

^b^ARIA-E rating scale 28

Injection site erythema events were all mild to moderate in intensity. ARIA-E events were dose- and *APOE* ε4 genotype-dependent and occurred rarely in the placebo arm. ARIA-H were also *APOE* ε4 genotype-dependent and more frequent with gantenerumab treatment (Table 6). ARIA-E occurred most frequently between 3 and 6 months of treatment; the incidence notably decreased after the first 9 months of treatment, and ARIA-E did not commonly recur (recurrence rate 13.8%) in patients who restarted treatment at half the original dose. The large majority of ARIA-E events (> 80%) were asymptomatic and resolved with dose adjustment. The most commonly reported symptom was headache.

## Discussion

In the SCarlet RoAD study, we investigated the effects of 105 mg or 225 mg of SC gantenerumab or placebo given every 4 weeks in patients with prodromal AD. A preplanned futility analysis was conducted on the primary endpoint (CDR-SB change from baseline) when ~ 50% of the patients had completed 2 years of treatment. No differences were observed in primary or secondary efficacy measures across the placebo and treatment arms, and the futility model predicted a < 6% chance of a positive study, which prompted the sponsor to discontinue dosing. These clinical outcomes in prodromal AD are reminiscent of phase III trials in mild to moderate AD with another antiaggregated Aβ antibody, bapineuzumab, in which investigators explored a limited dose range [21, 22]. Several factors were examined for their contributions to a negative outcome in SCarlet RoAD, including inactive compound, insufficient dosing, short trial duration, insensitive endpoints, and the population studied.

Despite the lack of a clinical benefit in the overall population, gantenerumab showed evidence of biological activity at the doses tested. Reduction of amyloid load as measured by PET was dose-dependent, indicative of successful target engagement in the brain and a confirmation of gantenerumab’s mechanism of action in a larger dataset and in a population at an earlier stage of the disease than in the earlier report [8]. Furthermore, gantenerumab treatment was associated with a dose-dependent reduction in CSF biomarkers that are thought to reflect core pathological processes of AD, including hyperphosphorylation of tau (p-tau), and downstream processes of neuronal and axonal degeneration (t-tau) [23], as well as synaptic dysfunction (neurogranin) [24].

Interestingly, in a subgroup of fast progressors, numerical differences in decline between treatment groups as measured by the ADAS-Cog 13 were observed, suggesting a dose-dependent clinical response. Similar dose-dependent trends were observed for MMSE and CANTAB, although not for CDR-SB. In contrast, no such effects were seen in patients predicted to have slow progression.

In SCarlet RoAD, gantenerumab doses of 105 and 225 mg SC every 4 weeks had been chosen to minimize the risk of ARIA in this mildly affected prodromal AD population. Dose- and *APOE* ε4 genotype-dependent ARIA were observed, as was injection site erythema. However, most of the ARIA were asymptomatic and rated by the investigators as mild or moderate, and most patients could continue dosing, albeit at a lower (halved) dose, with a low rate of ARIA-E recurrence.

It is hypothesized that higher doses of gantenerumab that result in more amyloid load reduction may be more likely to result in clinically meaningful efficacy. Recent phase Ib study results with aducanumab, an antibody with a preclinical profile similar to that of gantenerumab which was tested at higher doses in a similar population to the one enrolled in SCarlet RoAD, showed substantially greater and equally dose-dependent amyloid load reduction as measured by PET but also a higher incidence of ARIA [25]. The reported phase Ib results with aducanumab are suggestive of dose-dependent clinical effects; however, the sample was small.

The apparent dose-dependent clinical effect in fast progressors, combined with the biomarker outcomes in this study with gantenerumab, as well as the positive results of the phase Ib trial for aducanumab [25], suggest that gantenerumab may have been dosed too low to achieve substantial clinical benefit. Achieving higher exposures of gantenerumab while minimizing ARIA rates is expected to be important for a balanced benefit-risk profile. Because ARIA in SCarlet RoAD generally occurred within the first 6 months of dosing, with potentially higher frequency and/or severity at higher doses, it is hypothesized that using titration schemes to achieve high doses may result in substantial brain amyloid reduction that may be clinically beneficial yet minimize both the rate and severity of ARIA events [26]. To test this hypothesis, multiple dose titration regimens are being examined for their ability to minimize ARIA events at higher doses in open-label extensions of the ongoing gantenerumab trials. Whether treatment with an antibody such as gantenerumab at a higher dose provides not only a higher degree of brain amyloid reduction but also a clinical benefit must be demonstrated in randomized, placebo-controlled phase III clinical trials.

It has been questioned whether it is possible to observe clinically meaningful effects in prodromal AD populations within a 2-year study duration. Although negative phase III trials of anti-Aβ antibodies in mild and moderate AD have been 18 months long [21, 27], the phase Ib study results with aducanumab mentioned above suggest that clinical effects in an AD population at an earlier stage of the disease can already be identified after 12 months [25]. Therefore, the 2-year study duration in SCarlet RoAD was likely sufficient to show clinically meaningful effects.

The sensitivity of endpoints to effectively capture change in prodromal AD may potentially contribute to negative trial outcomes in this population. The primary endpoint in SCarlet RoAD was change in CDR-SB score over 2 years, in alignment with guidance from health authorities [28, 29] and other groups [30]. Recent analysis of the CDR-SB demonstrated test-retest reliability, construct validity to functional change, and sensitivity to decline in AD [31, 32]. SCarlet RoAD was the first global phase III study in prodromal AD using amyloid screening. The decline observed in the placebo group was slightly less (1.60 CDR-SB points over 2 years) than expected on the basis of ADNI data (1.92 CDR-SB points over 2 years) available at the time at which the study was designed. However, in another randomized clinical trial in CSF biomarker-supported prodromal AD, the decline in mean CDR-SB points over 2 years (placebo group) was reported to be 1.65 [33]. Hence, the observed decline in CDR-SB is consistent with another recent clinical trial. Other, older reports [34] had suggested a higher placebo decline, but these were observational studies and had other important differences in terms of study design and patient characteristics. The change from baseline in CDR-SB was consistent with changes observed in other measures of cognition (ADAS-Cog 13, MMSE) and function (FAQ). The sensitivity of CDR-SB to drug effects has also been suggested by the aducanumab phase Ib study, as mentioned above. Overall, the choice of CDR-SB as the primary endpoint seemed appropriate for the population examined. The observed rate of decline must be carefully considered in any sample size estimation for future studies if the same population is to be enrolled.

This report has important limitations. The study was stopped early for futility. All biomarker and clinical signals described resulted from exploratory analyses, some of which were conducted post hoc. Therefore, all analyses presented here, including *p* values, should be considered as strictly descriptive and hypothesis-generating, but not confirmatory.

## Conclusions

In summary, although the SCarlet RoAD study was stopped early for futility, dose-dependent effects observed in exploratory analyses on select clinical and biomarker endpoints suggest that higher dosing with gantenerumab may be necessary to achieve clinical efficacy. Exploratory analyses in a fast progressor subgroup were also suggestive of a dose-dependent effect of gantenerumab on some clinical endpoints. Growing evidence of the manageability of ARIA supports the investigation of higher doses of gantenerumab in subsequent clinical trials. Further phase III clinical trials employing dose titration schemes designed to mitigate the increased rate of ARIA-E expected at higher doses are planned to assess the degree of amyloid reduction by gantenerumab at these doses and to confirm whether such effects are associated with clinical benefits.

## Additional file

Additional file 1: List of institutional review boards and independent ethics committees. (DOCX 20 kb)

## Abbreviations

Aβ: Amyloid-beta
AD: Alzheimer’s disease
ADAS-Cog 13: Alzheimer’s Disease Assessment Scale–Cognitive subscale
ADNI: Alzheimer’s Disease Neuroimaging Initiative
APOE: Apolipoprotein E
ARIA: Amyloid-related imaging abnormalities
ARIA-E: Amyloid-related imaging abnormalities–edema
ARIA-H: Amyloid-related imaging abnormalities–microbleeds
CANTAB: Cambridge Neuropsychological Test Automated Battery
CDR-SB: Clinical Dementia Rating Sum of Boxes
AChE: Acetylcholinesterase
CSF: Cerebrospinal fluid
FAQ: Functional Activities Questionnaire
FCSRT: Free and Cued Selective Reminding Test
GCP: Good clinical practice
GDS: Geriatric Depression Scale
IDMC: Independent data monitoring committee
IV: Intravenous
MCI: Mild cognitive impairment
MMRM: Mixed-effects model repeated measurement
MMSE: Mini Mental State Examination
MRI: Magnetic resonance imaging
NPI-Q: Neuropsychiatric Inventory Questionnaire
PET: Positron emission tomography
p-tau: Phosphorylated tau 181
SC: Subcutaneous
SUVr: Standardized uptake value ratio
t-tau: Total tau

## References

[1] Bateman RJ, Xiong C, Benzinger TL, Fagan AM, Goate A, Fox NC (2012). Clinical and biomarker changes in dominantly inherited Alzheimer’s disease. N Engl J Med.

[2] Haass C, Selkoe DJ (2007). Soluble protein oligomers in neurodegeneration: lessons from the Alzheimer’s amyloid beta-peptide. Nat Rev Mol Cell Biol.

[3] Blennow K, de Leon MJ, Zetterberg H (2006). Alzheimer’s disease. Lancet.

[4] Dubois B, Feldman HH, Jacova C, DeKosky ST, Barberger-Gateau P, Cummings J (2007). Research criteria for the diagnosis of Alzheimer’s disease: revising the NINCDS-ADRDA criteria. Lancet Neurol.

[5] Sperling RA, Aisen PS, Beckett LA, Bennett DA, Craft S, Fagan AM, et al. Toward defining the preclinical stages of Alzheimer’s disease: recommendations from the National Institute on Aging-Alzheimer’s Association workgroups on diagnostic guidelines for Alzheimer’s disease. Alzheimers Dement. 2011;7(3):280–92. 10.1016/j.jalz.2011.03.003 PMC3220946 21514248

[6] Olsson B, Lautner R, Andreasson U, Ohrfelt A, Portelius E, Bjerke M (2016). CSF and blood biomarkers for the diagnosis of Alzheimer’s disease: a systematic review and meta-analysis. Lancet Neurol.

[7] Bard F, Cannon C, Barbour R, Burke RL, Games D, Grajeda H (2000). Peripherally administered antibodies against amyloid beta-peptide enter the central nervous system and reduce pathology in a mouse model of Alzheimer disease. Nat Med.

[8] Ostrowitzki S, Deptula D, Thurfjell L, Barkhof F, Bohrmann B, Brooks DJ (2012). Mechanism of amyloid removal in patients with Alzheimer disease treated with gantenerumab. Arch Neurol.

[9] Rosen WG, Terry RD, Fuld PA, Katzman R, Peck A (1980). Pathological verification of ischemic score in differentiation of dementias. Ann Neurol.

[10] Morris JC (1993). The Clinical Dementia Rating (CDR): current version and scoring rules. Neurology.

[11] Fleisher AS, Chen K, Liu X, Roontiva A, Thiyyagura P, Ayutyanont N (2011). Using positron emission tomography and florbetapir F18 to image cortical amyloid in patients with mild cognitive impairment or dementia due to Alzheimer disease. Arch Neurol.

[12] Rowe CC, Ackerman U, Browne W, Mulligan R, Pike KL, O’Keefe G (2008). Imaging of amyloid beta in Alzheimer’s disease with 18 F-BAY94-9172, a novel PET tracer: proof of mechanism. Lancet Neurol.

[13] Freeborough PA, Fox NC (1997). The boundary shift integral: an accurate and robust measure of cerebral volume changes from registered repeat MRI. IEEE Trans Med Imaging.

[14] Leung KK, Clarkson MJ, Bartlett JW, Clegg S, Jack CR, Weiner MW (2010). Robust atrophy rate measurement in Alzheimer’s disease using multi-site serial MRI: tissue-specific intensity normalization and parameter selection. Neuroimage.

[15] Bittner T, Zetterberg H, Teunissen CE, Ostlund RE, Militello M, Andreasson U (2016). Technical performance of a novel, fully automated electrochemiluminescence immunoassay for the quantitation of beta-amyloid (1-42) in human cerebrospinal fluid. Alzheimers Dement.

[16] Davidsson P, Blennow K (1998). Neurochemical dissection of synaptic pathology in Alzheimer’s disease. Int Psychogeriatr.

[17] Zetterberg H, Blennow K (2015). Neurogranin levels in cerebrospinal fluid: a new addition to the Alzheimer disease diagnostic toolbox. JAMA Neurol.

[18] Kvartsberg H, Duits FH, Ingelsson M, Andreasen N, Ohrfelt A, Andersson K (2015). Cerebrospinal fluid levels of the synaptic protein neurogranin correlates with cognitive decline in prodromal Alzheimer’s disease. Alzheimers Dement.

[19] Barkhof F, Daams M, Scheltens P, Brashear HR, Arrighi HM, Bechten A (2013). An MRI rating scale for amyloid-related imaging abnormalities with edema or effusion. AJNR Am J Neuroradiol.

[20] Delor I, Charoin JE, Gieschke R, Retout S, Jacqmin P (2013). Modeling Alzheimer’s disease progression using disease onset time and disease trajectory concepts applied to CDR-SOB scores from ADNI. CPT Pharmacometrics Syst Pharmacol..

[21] Salloway S, Sperling R, Fox NC, Blennow K, Klunk W, Raskind M (2014). Two phase 3 trials of bapineuzumab in mild-to-moderate Alzheimer’s disease. N Engl J Med.

[22] Vandenberghe R, Rinne JO, Boada M, Katayama S, Scheltens P, Vellas B (2016). Bapineuzumab for mild to moderate Alzheimer’s disease in two global, randomized, phase 3 trials. Alzheimers Res Ther.

[23] Blennow K, Hampel H, Weiner M, Zetterberg H (2010). Cerebrospinal fluid and plasma biomarkers in Alzheimer disease. Nat Rev Neurol.

[24] Kester MI, Teunissen CE, Crimmins DL, Herries EM, Ladenson JH, Scheltens P (2015). Neurogranin as a cerebrospinal fluid biomarker for synaptic loss in symptomatic Alzheimer disease. JAMA Neurol.

[25] Sevigny J, Chiao P, Bussiere T, Weinreb PH, Williams L, Maier M (2016). The antibody aducanumab reduces Aβ plaques in Alzheimer’s disease. Nature.

[26] Viglietta V, O’Gorman J, Williams L, Tian Y, Sandrock A, Doody R, Salloway S, Barkhof F (2016). Randomized, double-blind, placebo-controlled studies to evaluate treatment with aducanumab (BIIB037) in patients with early Alzheimer’s disease: phase 3 study design [abstract]. Neurology.

[27] Doody RS, Farlow M, Aisen PS, Alzheimer’s Disease Cooperative Study Data Analysis and Publication Committee (2014). Phase 3 trials of solanezumab and bapineuzumab for Alzheimer’s disease. N Engl J Med.

[28] European Medicines Agency (EMA). Discussion paper on the clinical investigation of medicines for the treatment of Alzheimer’s disease and other dementias. London: EMA; 23 Oct 2014. http://www.ema.europa.eu/docs/en_GB/document_library/Scientific_guideline/2014/10/WC500176827.pdf. Accessed 7 July 2017.

[29] Food and Drug Administration (FDA). Guidance for industry. Alzheimer’s disease: developing drugs for the treatment of early stage disease Silver Spring, MD: Center for Drug Evaluation and Research, FDA; Feb 2013. https://www.fda.gov/downloads/drugs/guidancecomplianceregulatoryinformation/guidances/ucm338287.pdf. Accessed 7 July 2017.

[30] Cedarbaum JM, Jaros M, Hernandez C, Coley N, Andrieu S, Grundman M (2013). Rationale for use of the Clinical Dementia Rating Sum of Boxes as a primary outcome measure for Alzheimer’s disease clinical trials. Alzheimers Dement.

[31] Coley N, Andrieu S, Jaros M, Weiner M, Cedarbaum J, Vellas B (2011). Suitability of the Clinical Dementia Rating-Sum of Boxes as a single primary endpoint for Alzheimer’s disease trials. Alzheimers Dement.

[32] Edgar C, Rylands A, Volz D, Mertes M, Gruendl E, Fontoura P, Santarelli L, Lasser R. Comparative traditional psychometrics of cognitive and functional endpoints in a prodromal Alzheimer’s disease population. Presented at Clinical Trials on Alzheimer’s Disease conference, Barcelona, Spain, November 2015

[33] Coric V, Salloway S, van Dyck CH, Dubois B, Andreasen N, Brody M (2015). Targeting prodromal Alzheimer disease with avagacestat: a randomized clinical trial. JAMA Neurol.

[34] Williams MM, Storandt M, Roe CM, Morris JC (2013). Progression of Alzheimer disease as measured by Clinical Dementia Rating Sum of Boxes scores. Alzheimers Dement.

