# Correction to: A phase III randomized trial of gantenerumab in prodromal Alzheimer’s disease

**DOI:** 10.1186/s13195-018-0409-4

**Published:** 2018-09-27

**Authors:** Susanne Ostrowitzki, Robert A. Lasser, Ernest Dorflinger, Philip Scheltens, Frederik Barkhof, Tania Nikolcheva, Elizabeth Ashford, Sylvie Retout, Carsten Hofmann, Paul Delmar, Gregory Klein, Mirjana Andjelkovic, Bruno Dubois, Mercè Boada, Kaj Blennow, Luca Santarelli, Paulo Fontoura, for the SCarlet RoAD Investigators

**Affiliations:** 1 Product Development, Neuroscience, Genentech Inc., South San Francisco, CA USA; 2 MedDay Pharmaceuticals, Boston, MA USA; 3 Formerly Roche Translational & Clinical Research Center, New York, NY USA; 4 VU University Medical Center, Amsterdam, The Netherlands; 5 Institute of Neurology, UCL, London, UK; 6 Roche Pharma Research and Early Development, NORD, Basel, Switzerland; 7 Roche Products Limited, Welwyn Garden City, UK; 8 Roche Pharma Research and Early Development, Clinical Pharmacology, Roche Innovation Center, Basel, Switzerland; 9 Clinical Pharmacology and Bioanalytical R&D, Pharmaceutical Sciences, Roche Pharma Research and Early Development, Roche Innovation Center Basel, Basel, Switzerland; 10 Alzheimer Institute and ICM, UMR-S975, Salpêtrière University Hospital, AP-HP, Pierre and Marie Curie University, Paris, France; 11 Research Center and Memory Clinic of Fundació ACE, Institut Català de Neurociències Aplicades, Barcelona, Spain; 12 Formerly Roche Pharma Research and Early Development, NORD, Basel, Switzerland

## Correction

Following publication of the original article [1], the authors reported errors in the formatting of the table. The details of the errors are as follows:

In Table 1, the data presented do not correspond to the row title:

- The education row currently contains incorrect data.
- The weight row currently contains the years of education.
- The APOEε4 genotype row currently contains the weight data.

In Table 1, the data should be displayed as follows:

Variable

Intention-to-treat population (*n* = 797)

Placebo

(*n* = 266)

Gantenerumab

105 mg

(*n* = 271)

Gantenerumab

225 mg

(*n* = 260)

Age, years, mean (SD)

69.5 (7.5)

70.3 (7.0)

71.3 (7.1)

Education, years, mean (SD)

12.6 (4.3)

12.9 (4.8)

12.1 (4.5)

Weight, kg, mean (SD)

69.8 (12.9)

70.5 (13.6)

70.1 (12.5)

APOEε4 genotype, %^a^
